# Echoes of stress: From molecular whispers to social thunderstorms

**DOI:** 10.1016/j.ynstr.2025.100766

**Published:** 2025-10-24

**Authors:** Juan Pablo Lopez

**Affiliations:** Department of Neuroscience, Karolinska Institutet, Stockholm, Sweden

**Keywords:** Early-life adversity, Resilience, Susceptibility, Sex differences, Treatment resistance, Translational neuroscience

## Abstract

Stress is often described as a fleeting feeling, a momentary surge of tension or anxiety, but in my work as a neurobiologist, I have come to understand it as something far more enduring and complex. It is not merely a reaction to external pressure, it is a biological echo that resonates through our cells, brain circuits, and peripheral systems, ultimately shaping behavior and health. These echoes begin as molecular whispers, subtle shifts in hormonal regulation, gene expression, epigenetic marks, and synaptic plasticity, but over time, they can build into thunderstorms, manifesting as psychiatric and other stress-related disorders. My research has focused on detecting and translating these echoes into meaningful biological insight. Through this perspective article, I describe how early-life adversity, sex differences, and individual variability shape susceptibility and resilience. It highlights the promise of precision tools, from single-cell technologies to AI-driven behavioral tracking, to decode stress in motion and across systems. Through studies spanning human molecular genetics, animal models, and systems neuroscience, I envision a future of stress research that embraces biological complexity, prioritizes translational relevance, and aspires to personalize mental health care by decoding the molecular and circuit-level biology of lived experience.

## 1 Early whispers, lifelong storms: the imprint of childhood adversity

One of the most formative insights in my career has been understanding how early-life adversity (ELA), particularly during critical developmental windows, can program long-lasting changes in the brain (Birnie and Baram, 2025). Experiencing significant adversity in early life does not just disrupt development, it actively reshapes it. For example, we have shown how ELA can shape social subordination later in life and drive cell type–specific transcriptomic changes in the brains of mice (Kos et al., 2023; van et al., 2025). These findings reinforce our understanding that the brain, particularly during childhood and adolescence, is highly plastic and sensitive to environmental input. Stress during these periods can recalibrate core stress-regulatory systems, such as the hypothalamic-pituitary-adrenal (HPA) axis, and leave enduring molecular marks on stress-related genes and neural circuits (Kos et al., 2023). These neurobiological scars, lasting molecular and circuit-level imprints left by early adversity, can influence how individuals respond to stress later in life. Much like physical scars, they may serve as reminders of past challenges while also reflecting the brain's capacity to heal and adapt. In some cases, these biological scars may increase vulnerability to psychiatric disorders. In others, they may foster resilience by encoding adaptive recalibration and recovery mechanisms (Torres-et al., 2025). I see our early life experiences, not as a fixed determinant of fate, but as a dynamic process shaped by the interplay of developmental timing, genetic predisposition, and environmental context. Looking ahead, I believe the field of stress neurobiology must prioritize longitudinal, multimodal research that captures how stress-sensitive circuits adapt and evolve over time. This includes mapping trajectories at both the micro level (e.g., gene expression and epigenetic states) and the macro level (e.g., complex social behavior and cognitive outcomes) (Miranda et al., 2023; Gururajan et al., 2018). By identifying the molecular “switches” that regulate adaptive versus maladaptive responses, we can develop targeted interventions (e.g., behavioral, pharmacological, or neuromodulatory) that buffer or even reverse the long-term effects of early-life adversity and trauma. The implications are profound. If we can detect the molecular fingerprints of early life adversity, we can create age-specific diagnostics and interventions aligned with the brain's developmental trajectory. This opens the door to early screening tools capable of identifying children at heightened risk, enabling timely and personalized support before symptoms emerge. It is a paradigm shift: from reactive to preventive psychiatry, catching the echoes of stress before they escalate into storms.

## 2 Two pathways through the same storm: sex differences in stress neurobiology

A key realization in my work has been that stress is processed and expressed differently across sexes, with distinct biological consequences at the molecular and behavioral levels. While foundational research over the past few decades has firmly established sex differences in stress systems, recent advances in transcriptomics and circuit-level analyses are revealing new, cell-type–specific mechanisms that deepen our understanding of these differences. This evolving molecular resolution challenges the long-standing assumption that stress mechanisms are largely universal and underscores the need for a sex-aware neurobiology field (Bale and Epperson, 2015). We now know that the molecular signatures of stress can be sex-specific and translate into distinct behavioral outcomes (Brivio et al., 2020). In line with emerging research, our recent studies show that sex influences the brain's transcriptional response to stress, with females displaying distinct regulation of genes related to stress reactivity, neuroinflammation, and hormonal signaling, pathways that are either absent or differently regulated in males (Kos et al., 2023; van et al., 2025; Brivio et al., 2023; van et al., 2023). These differences appear as cell-type-specific molecular signatures across key stress-regulatory regions, with oligodendrocytes and certain neuronal subtypes emerging as critical mediators. These findings underscore the need for a sex-aware neurobiology field, one that treats sex not as a statistical nuisance, but as a fundamental biological variable. Looking forward, we must build comprehensive, sex-specific atlases of stress-related gene expression across brain regions and developmental stages. We need to understand how hormonal cycles interact with stress circuits and how these interactions influence susceptibility to disorders like anxiety, depression, and other stress-related disorders. These insights are not only scientifically urgent, but they are also clinically essential. Many psychiatric medications have been developed and tested predominantly in male animals and patients, leading to treatments that may be less effective, or even harmful, for women (Keers and Aitchison, 2010; LeGates et al., 2019). By identifying sex-specific molecular targets, we can design therapies that are more precise, more effective, and more equitable. Ultimately, I envision a future where diagnostics and treatments are tailored to the individual, their biological sex, and developmental history. In the broader narrative of stress, acknowledging and addressing sex differences is a crucial step toward transforming echoes of susceptibility into pathways of resilience.

## 3 A molecular compass to navigate stress adaptation: resilience and susceptibility

As we deepen our understanding of stress neurobiology, the dichotomy between susceptibility and resilience emerges as a central axis for future research (Nestler and Russo, 2024). Advances in decoding the molecular signatures of stress, particularly those involving circuit-level plasticity, epigenetic modifications, and neuroimmune interactions, reveal how stress embeds itself on the brain (Akil and Nestler, 2023). Resilience is not a static trait but a process of adaptive recalibration, involving protective mechanisms such as neurotrophic support, efficient stress hormone regulation, and social buffering (Franklin et al., 2012). Susceptibility, while also dynamic, reflects a trajectory shaped by early-life experiences, genetic predispositions, and environmental exposures that may lead to maladaptive outcomes. Both processes are context-dependent and reflect the brain's capacity to respond to stress in divergent ways, toward recovery or vulnerability. Alongside other studies, our work has shown that different animal models, developmental timepoints, and biological variables such as sex can shape the molecular signatures of resilience and susceptibility to stress through distinct, cell-type-specific pathways (van et al., 2023; Lopez et al., 2021). These molecular whispers, subtle shifts in gene expression, and synaptic remodeling, can predispose individuals to maladaptive outcomes. To truly understand how stress shapes behavior and health, we must adopt a systems-level approach, one that integrates circuit-level functional manipulations with single-cell technologies (e.g., transcriptomics, epigenomics, proteomics), and high-resolution behavioral phenotyping (Gururajan et al., 2018). We need more advanced computational models capable of predicting how molecular-level changes cascade into circuit dynamics and behavioral outcomes. Most importantly, these models must be rigorously validated across species, from animal models to human datasets. This framework invites us to rethink treatment. Rather than merely dampening symptoms, we could aim to rewire the brain's response to stress, rewriting the molecular narrative left by past experiences. This could revolutionize how we approach chronic stress, trauma, and treatment-resistant depression. The future of stress neurobiology lies in bridging molecular, behavioral, and societal domains. We must invest in longitudinal, cross-cultural studies that capture the full spectrum of stress experiences. Precision psychiatry, informed by biomarkers of susceptibility and resilience, could transform how we predict and prevent stress-related disorders. In this unfolding narrative, stress is both a whisper and a storm. Our task is not only to decode its signals, but to amplify the voices of resilience.

## 4 Harnessing the storm: toward precision and treatment response

Optimizing antidepressant treatment remains one of the most urgent challenges in neuropsychiatric research today (Howes et al., 2022). Despite decades of pharmacological development, many individuals with stress-related disorders fail to respond to first-line treatments (Paul and Potter, 2024). This therapeutic gap reflects our incomplete understanding of how stress and trauma reshape the brain, from molecular whispers to behavioral storms. Stress leaves behind molecular echoes, imprints on gene expression, synaptic plasticity, and neuroimmune signaling, that influence how individuals respond to treatment (Willner et al., 2013). These echoes are shaped by developmental history, genetic background, and environmental exposures. For example, in my early work, I demonstrated that specific biological markers, such as microRNAs and epigenetic modifications, hold promise as predictive indicators of individual variability in antidepressant treatment response (Lopez et al., 2013, 2014, 2017, 2018; Cruceanu et al., 2017). I am a firm believer that variability in treatment response is not noise, it is signal. It tells us we must move beyond one-size-fits-all approaches and toward precision strategies that account for individual stress histories and backgrounds. Future research must prioritize identifying biomarkers that predict antidepressant response (Kennis et al., 2020). This includes integrating multi-omics data with neuroimaging and behavioral phenotyping (Miranda et al., 2023). The goal is not just to know who will respond, but why. What molecular scars has stress left behind? Which brain circuits have been sensitized or silenced? And how can we target these changes to restore adaptive function? We must also redefine treatment success. Rather than simply alleviating symptoms, we should aim to recalibrate the body's stress response systems. Embracing novel approaches, such as rapid-acting antidepressants and neuromodulation offer promise, but their clinical integration must be guided by mechanistic insight (Liao et al., 2025; Krystal et al., 2024). The echoes of stress extend beyond the brain. They resonate through the body, the immune system, and the social world. Optimizing treatment requires a broader lens, one that considers social determinants of stress and systemic barriers to care. Community-based interventions, digital therapeutics, and culturally sensitive models must be part of the solution.

## 5 Decoding the language of stress in motion: precision tracking in behavioral neuroscience

In neuropsychiatry, we are moving beyond reductionist models to embrace the full complexity of behavior in its natural context (Shemesh and Chen, 2023). A holistic understanding of stress neurobiology demands ecologically valid and technologically advanced approaches to behavioral genotyping (Bordes et al., 2023). Stress is not a static event but a dynamic, multilayered process across molecular, neural, behavioral, and social domains (von Mucke et al., 2023). Traditional paradigms have often examined these layers in isolation, limiting our grasp of stress manifestations. Today, high-throughput behavioral tracking, machine learning-based ethology, and integrative neurotechnologies allow us to capture the “behavioral whispers” of stress as they ripple into complex behavioral and social storms (Miranda et al., 2023). A particularly exciting frontier is the rise of precision tracking systems to decode the behavioral language of individuals with unprecedented resolution. Thanks to recent innovations and implementation of modern tools like DeepLabCut (Mathis et al., 2018) and SLEAP (Pereira et al., 2022), we can now track multiple animals in social environments, identify individuals without invasive markers, and extract rich behavioral motifs using supervised and unsupervised methods. Using the Social Box system developed by Prof. Alon Chen, now implemented in my lab, we have demonstrated how these tools enable in-depth investigations of pharmacological and genetic interventions by allowing both short- and long-term, continuous tracking of socially relevant behaviors with minimal experimental interference (Kos et al., 2023; Shemesh and Chen, 2023; Lopez et al., 2022; Shemesh et al., 2013; Anpilov et al., 2025; Forkosh et al., 2019). These technologies reduce labor intensity, minimize experimental bias, enhance standardization, and allow animals to be studied in naturalistic environments, capturing complex group dynamics and long-term behavioral patterns across circadian cycles. They detect subtle, stress-relevant behaviors, from antagonistic actions to pro-social behaviors and interactions with an enriched environment. Looking ahead, the field must prioritize context-aware behavioral genotyping frameworks that reflect naturalistic behavior. Integrating wearable biosensors, closed-loop neural modulation, and AI-driven behavioral prediction models is crucial. These innovations must be guided by ethical foresight to ensure precision aligns with compassion, ethical integrity, and a commitment to animal welfare. Ultimately, these methods are a gateway to deeper understanding. Aligning high-resolution behavioral phenotyping with state-of the-art molecular and circuit-level analyses brings us closer to identifying the precise neurobiological mechanisms governing stress responses (Miranda et al., 2023). This is at the core of our research: uncovering the molecular underpinnings of stress to better predict, prevent, and treat neuropsychiatric disorders. In listening to the full echo of stress, from its quietest behavioral signals to its loudest social aftershocks, we carve a path toward a more integrated, precise, and impactful science of the mind.

## 6 Listening to the Brain's smallest voices: from single cells to whole systems

In stress neurobiology, we are entering an era where the once faint molecular whispers of stress can now be heard with remarkable clarity. Advances in molecular technologies allow us to resolve the stress response at the level of individual cells, circuits, and systems (Miranda et al., 2023; Gururajan et al., 2018). This shift from broad strokes to fine-grained resolution is transforming our understanding and intervention in stress-related disorders. Single-cell technologies like RNA sequencing (Aldridge and Teichmann, 2020), spatial transcriptomics (Lein et al., 2017), and emerging epigenomics (Lu et al., 2023) and proteomics (Bennett et al., 2023) can help pinpoint how neurons, glial, and vascular cells react to stressors, uncovering cell-type-specific vulnerabilities and adaptive responses (Miranda et al., 2023). For example, we have applied some of these methods to identify the molecular signatures of stress- and treatment-responsive cells, and to track how these responses evolve over time and across distinct brain regions (Kos et al., 2023; Brivio et al., 2020; Lopez et al., 2021). In addition, activity sensors and genetically encoded indicators enable real-time monitoring of specific brain cells and circuits during stress exposure (Kawashima et al., 2014; Wu et al., 2022). They offer a dynamic view of how stress is processed and regulated within the brain, revealing the functional contributions of distinct neuronal populations (Miranda et al., 2023; Gururajan et al., 2018). This integration of structure and function builds a mechanistic understanding of how stress alters brain circuitry. Expanding this view, stress is a whole-body phenomenon involving interactions between the central nervous system, peripheral organs, and the immune and endocrine systems (Miranda et al., 2023). Future directions must embrace this systems-level perspective. Technologies that integrate brain and body signals will capture the full physiological landscape of the stress response. Our goal is to understand stress more deeply. By increasing molecular resolution across scales, we identify the precise biological mechanisms underlying susceptibility and resilience. This transforms our understanding of stress from a fragmented puzzle into a coherent narrative. The future of stress neurobiology lies in distilling the echoes of stress into knowledge and meaningful action.

## 7 When the brain whispers, the body listens: the neurobiology of stress across systems

In stress neurobiology, we now understand much better how chronic stress affects far more than our brains and mental health, it disrupts the entire body and is a risk factor for a wide range of diseases and medical conditions (Miranda et al., 2023). The brain does not suffer in isolation; it communicates with the body through molecular signals, hormones, and immune responses (McEwen, 2017). Once seen as a psychological burden, chronic stress is now understood as a systemic disruptor, capable of altering physiology from cells to organ systems. Recent advances reveal how stress-induced brain changes reverberate throughout the body (Roberts and Karatsoreos, 2021). Glucocorticoids, inflammatory cytokines, and autonomic signals translate psychological strain into physical consequences, increasing the risk for cardiovascular disease, metabolic disorders, immune dysfunction, and even accelerated aging (Godoy et al., 2018). In addition, emerging research on the gut-brain axis further underscores how microbial communities in the gastrointestinal tract can influence stress reactivity, neuroinflammation, and even behavior, adding a new layer of complexity to the systemic nature of stress (Cryan et al., 2019; Sanz et al., 2025). Based on all this, we now understand better that stress is not just a mental state, it is a full-body experience with profound health implications. This broader view demands integrative, cross-disciplinary approaches that unite neurobiology, immunology, endocrinology, and systems biology (Miranda et al., 2023). Tools like multi-omics profiling, biomarker tracking, and brain-body imaging are essential for mapping the bidirectional pathways linking stress to disease. Understanding why some individuals are more resilient than others will require personalized models that consider genetic, environmental, and psychosocial factors. As someone deeply engaged in this field, I believe the future of stress research lies in decoding these complex interactions with both precision and compassion. We must listen not only to the brain's molecular whispers but also to the physiological echoes that ripple through the body. Only then can we design interventions as interconnected as the systems they aim to heal.

## 8 Conclusion: riding the storm

One of the guiding principles of my lab is that basic science should inform clinical practice, and vice versa. That is why we have built a multidisciplinary framework that integrates molecular biology, behavioral neuroscience, and computational modeling. We collaborate closely with clinicians and data scientists to ensure that our findings are not only scientifically rigorous, but also actionable in real-world settings. Looking ahead, I see several key priorities for the field of stress neurobiology. First, we must move beyond static models and embrace the temporal dimension of stress. Stress is not a snapshot, it's a process. To truly understand it, we need to study how stress responses unfold over time, how they interact with developmental milestones, and how they are shaped by prior experiences. This calls for longitudinal studies, time-sensitive biomarkers, and interventions tailored to specific developmental windows. Second, we must prioritize diversity in our models, our methods, and our populations. This includes accounting for sex differences, genetic variability, environmental context, and cultural background. Psychiatric disorders are heterogeneous, and our research must reflect that complexity. By embracing diversity, we can develop more inclusive and effective treatments. Third, we need to shift our focus from vulnerability to resilience. Much of stress research has understandably centered on what goes wrong, but we also need to understand what goes right. Why do some individuals thrive despite adversity? What molecular mechanisms underlie resilience? And how can we harness these mechanisms to promote mental health? I believe resilience is not merely the absence of pathology, but the presence of adaptive plasticity, and that it can be cultivated through targeted interventions. As we move forward, the next generation of researchers must prioritize integrative and longitudinal approaches that bridge molecular insights with complex social dynamics, ensuring that our science not only decodes stress, but also transforms lives.

The title of this perspective article “*Echoes of Stress: From Molecular Whispers to Social Thunderstorms*” captures the arc of my young scientific journey. I have spent my career listening to the brain's whispers: the subtle changes in gene expression, the quiet shifts in cell activity, the faint traces of past experiences embedded into the epigenome. But I have also witnessed how these whispers can grow louder, how they accumulate, amplify, and erupt into the storms we recognize as psychiatric illness, that if left unaddressed, can lead to devastating consequences, including loss of life. My goal is to interrupt that process, to catch the echoes before they become storms. To understand the biology of stress not just to treat disease, but to promote health. And yet, it is important to remember that stress is not inherently harmful. In its acute form, stress is a vital biological signal, mobilizing energy, sharpening focus, and enhancing adaptation. The challenge for the future is not to eliminate stress, but to understand its dual nature: to distinguish the whispers that foster ease and resilience from those that strengthen the storms that harm. To find a balance between harm and ease. This is the future I'm working toward, a future where neurobiology is preventive, personalized, and profoundly human. Stress is not just a challenge, but it is an opportunity. An opportunity to understand the brain in motion, to intervene before pathology takes hold, and to promote mental health in a way that is grounded in biology and responsive to experience. That is the future I'm committed to building, one molecular whisper at a time.

## Funding

JPL receives research funding from the Swedish Society for Medical Research (SSMF), the Swedish Research Council (VR), the Swedish Brain Foundation (Hjärnfonden), the Strategic Research Area Neuroscience (StratNeuro), and the European Research Council (ERC) through a Starting Grant.

## Declaration of competing interest

The authors declare that they have no known competing financial interests or personal relationships that could have appeared to influence the work reported in this paper.

**Figure:**
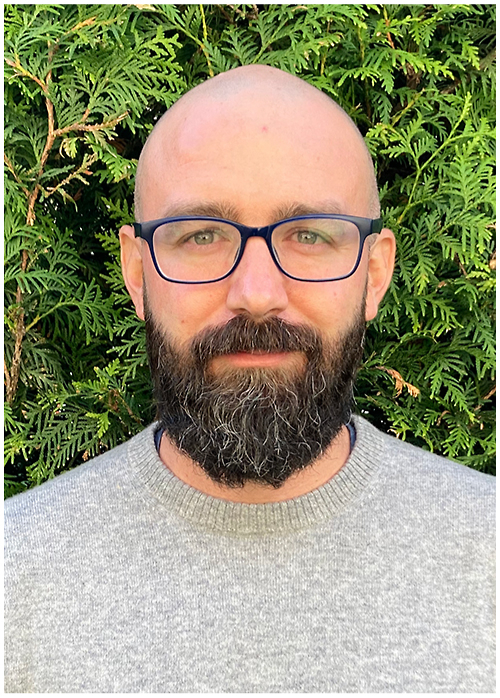

## Data Availability

No data was used for the research described in the article.
